# Interfacial Electronic Charge Trapping and Photonic Carrier Excitation Coupling in Solution-Processed Zinc–Tin Oxide Thin-Film Transistors Applied for Logic Gate Design and Quantized Neural Network

**DOI:** 10.1021/acsami.4c15102

**Published:** 2024-12-18

**Authors:** Pei-Hsuan Chang, Wun-Yun Lin, Ya-Chi Huang, Yu-Chieh Chen, Li-Chung Shih, Jen-Sue Chen

**Affiliations:** † Department of Materials Science and Engineering, National Cheng Kung University, Tainan 70101, Taiwan; ‡ Academy of Innovative Semiconductor and Sustainable Manufacturing, National Cheng Kung University, Tainan 70101, Taiwan

**Keywords:** oxide thin-film transistors, phototransistor, solution process, logic circuit, quantized neural network

## Abstract

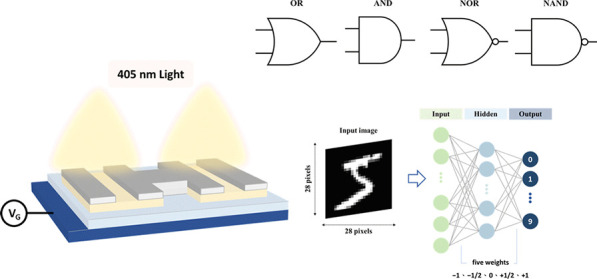

Components needed in Artificial Intelligence with a higher information capacity are critically needed and have garnered significant attention at the forefront of information technology. This study utilizes solution-processed zinc–tin oxide (ZTO) thin-film phototransistors and modulates the values of *V*_G_, which allows for the regulation of electron trapping/detrapping at the ZTO/SiO_2_ interface. By coupling the excited photonic carrier and electronic trapping, logic gates such as “AND,” “OR,” “NAND,” and “NOR” can be achieved. With the exponential growth in data generation, efficient processing and storage solutions are imperative. However, extensive data transfer between computing units and storage limits the level of artificial neural networks (ANNs). Consequently, quantized neural networks (QNNs) have gained interest for their reduced computational resource requirements and lower consumption. In this context, we introduce an optimized ternary logic circuit based on ZTO devices. By utilizing optical modulation to adjust the turn-on voltage of the single device, we demonstrate the achievement of ternary current states, thereby providing three distinct discrete states. This configuration can be extended to QNN computing, demonstrating multilevel quantized current values for in-memory computation. We achieved a handwriting digit recognition rate of 91.6%, thereby demonstrating reliable QNN hardware performance. This robust QNN performance indicates that the metal oxide phototransistor shows significant potential for future ternary computing systems.

## 1 Introduction

In recent years, a significant increase in data generation has necessitated the development of efficient processing and storage solutions. Traditionally, these demands have been primarily met through silicon-based CMOS (complementary metal-oxide-semiconductor) systems. However, as the need for faster data processing and larger storage capacities continues to grow, the limitations of CMOS scaling have become increasingly apparent. These limitations include power consumption, computational speed, and device density, all exacerbated by complex circuit design.^1−6^ Furthermore, the binary logic system inherent in CMOS technology restricts the number of logic states achievable per device unit, posing challenges for enhancing device and interconnect density.

However, due to the inherently binary nature of CMOS transistors, which possess only “ON” and “OFF” states, achieving more logic states necessitates a larger number of transistors.^7^ Systems with more than two logic states have attracted considerable attention due to their potential to significantly enhance computational efficiency. Some researchers have proposed systems with multiple logic states. For example, ternary transistors, characterized by three distinct states (0, 1, and 2), represent a fundamental element of such systems.^8−11^ In comparison to conventional binary state devices, ternary devices offer the advantage of reduced system complexity and enhanced energy efficiency by minimizing the requisite number of transistors and interconnections. In the context of ternary transistors, the drain current operates within a range delineated by the off-state current (*I*_OFF_) and the on-state current (*I*_ON_), thereby establishing a stable current state corresponding to the state value “1” and facilitating the emergence of a double-valued threshold voltage.^12^

Artificial neural networks (ANNs) are extensively utilized across a broad spectrum of applications, including image classification, voice recognition, and natural language processing.^13−16^ These networks achieve high levels of accuracy through the employment of advanced computational units and substantial data sets. However, the application of ANNs within the domain of artificial intelligence of things (AIoT) is constrained by the limited computational power and the substantial energy consumption required for executing these algorithms.^17−19^ In response to these constraints, the quantized neural network (QNN) algorithm has been introduced as a viable solution for designing specialized neural engines that require reduced computational resources.^20^ By employing a limited number of digits to represent data, the QNN algorithm significantly reduces resource utilization compared to the traditional 32-bit full-precision weights used in ANN algorithms based on the von Neumann architecture, thereby enhancing computational efficiency.^21,22^

In this study, we fabricated zinc tin oxide thin-film phototransistors on a p^+^Si substrate by a solution process. The conductive properties of the device can be modulated through various modulation methods. Utilizing circuit composition, we constructed four reconfigurable logic gates: “AND,” “OR,” “NAND,” and “NOR.” These configurations were achieved by applying various combinations of optical and electrical signal inputs. Subsequently, we designed circuits in series to realize ternary transfer characteristics and enabled turn-on voltage modulation through gate biasing and photonic programming. Finally, based on these ternary characteristics, we established five balanced weight values (−1, – 1/2, 0, 1/2, and 1) for in-memory computing. We developed a QNN algorithm for image classification, incorporating a quantized neural processor with ternary characteristics into machine learning tasks and utilizing five balanced weight values. Using the MNIST image data set for image classification, the proposed method exhibited accuracy comparable to that of the full-precision approach.

## 2 Results and Discussion

**Figure 1:**
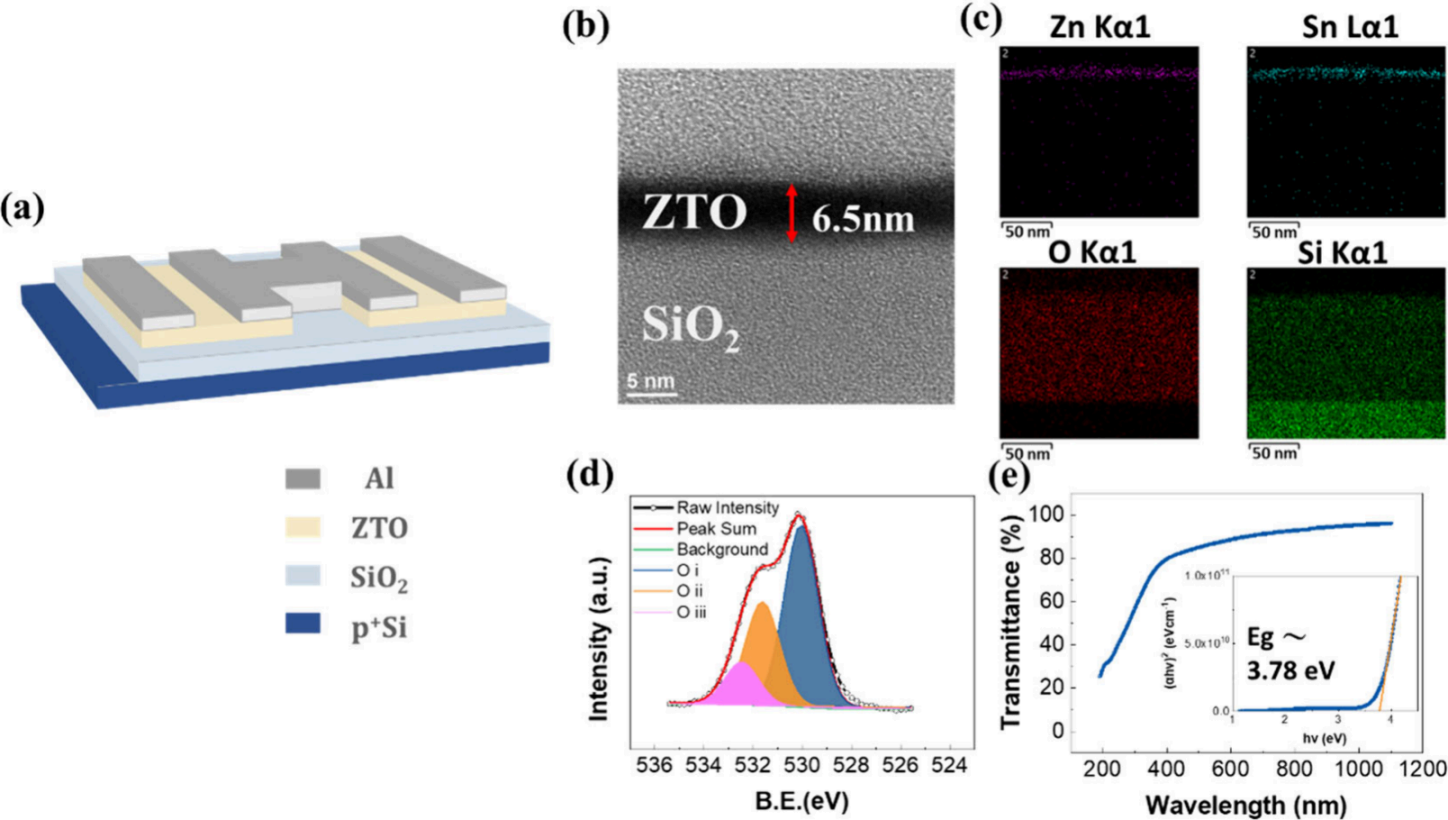
(a) A schematic illustration of two ZTO devices integrated in series on a SiO_2_/Si substrate. (b) Cross-sectional TEM image of the ZTO. (c) EDS elemental mapping analysis of the ZTO. (d) XPS spectra of O 1s of the ZTO film. (e) The UV–vis transmission spectrum of the ZTO film. The inset shows the corresponding plot of (α*h*υ)^2^ as a function of photon energy for determining the optical bandgap.

Figure 1a illustrates a schematic of the device, which used zinc–tin oxide (ZTO) as the channel material and p^+^Si as the gate electrode. In the device structure, two devices were connected in series, referred to as two serially integrated ZTO devices. However, the basic electrical measurements were performed using a single ZTO phototransistor device. For material characterizations, ZTO films were prepared on SiO_2_ by spin coating. Figure 1b shows the cross-sectional transmission electron microscopy (TEM) image of the ZTO film deposited on SiO_2_, revealing a thickness of approximately 6.5 nm. Additionally, an energy-dispersive spectrometer (EDS) was used to analyze the elemental distribution within the ZTO phototransistor, confirming the presence of Zn, Sn, O, and Si, as shown in Figure 1c.

The chemical bonding states of the ZTO film were analyzed through O 1s XPS, as illustrated in Figure 1d. The binding energies of various core level electrons were determined to account for the charging effect by using the adventitious C 1s peak at 284.6 eV as a reference. The O 1s peak was separated into three components: lattice oxygen in ZTO (O_I_), oxygen in proximity to oxygen deficient (O_II_), and oxygen originating from water molecules adsorbed on the ZTO surface (O_III_), with corresponding energies of 530, 531.6, and 532.4 eV, respectively.^23,24^ The relative proportions of O_I_/(O_total_), O_II_/(O_total_), and O_III_/(O_total_) were determined to be 55.46%, 31.45%, and 13.09%, respectively, indicating the significant role of oxygen vacancies in the subsequent measurements. Additionally, we calculated the atomic ratio of zinc, tin, and oxygen elements using XPS. The semiquantitative atomic ratio of an element is attained by dividing the peak area by the sensitivity factor, as equation S1 is shown in Supporting Information. The spectrum, integrated area, and sensitivity factor for each element are shown in the Figure S2 and Table S2. As a result, we confirmed that the semiquantitative atomic ratio of Zn:Sn:O was 1.3:1:2.7.

The UV–vis transmittance spectra of the ZTO films deposited on the quartz glass are shown in Figure 1e. The optical bandgap (*E*_g_), was analyzed using the Tauc plot method. The linear portion of the (α*h*ν)^2^ vs *h*ν plot, shown in the inset of Figure 1e, where α is the absorption coefficient and *h*ν is the incident photon energy, was used to estimate the *E*_g_ value. The estimated bandgap of the ZTO film was found to be 3.78 eV.

**Figure 2:**
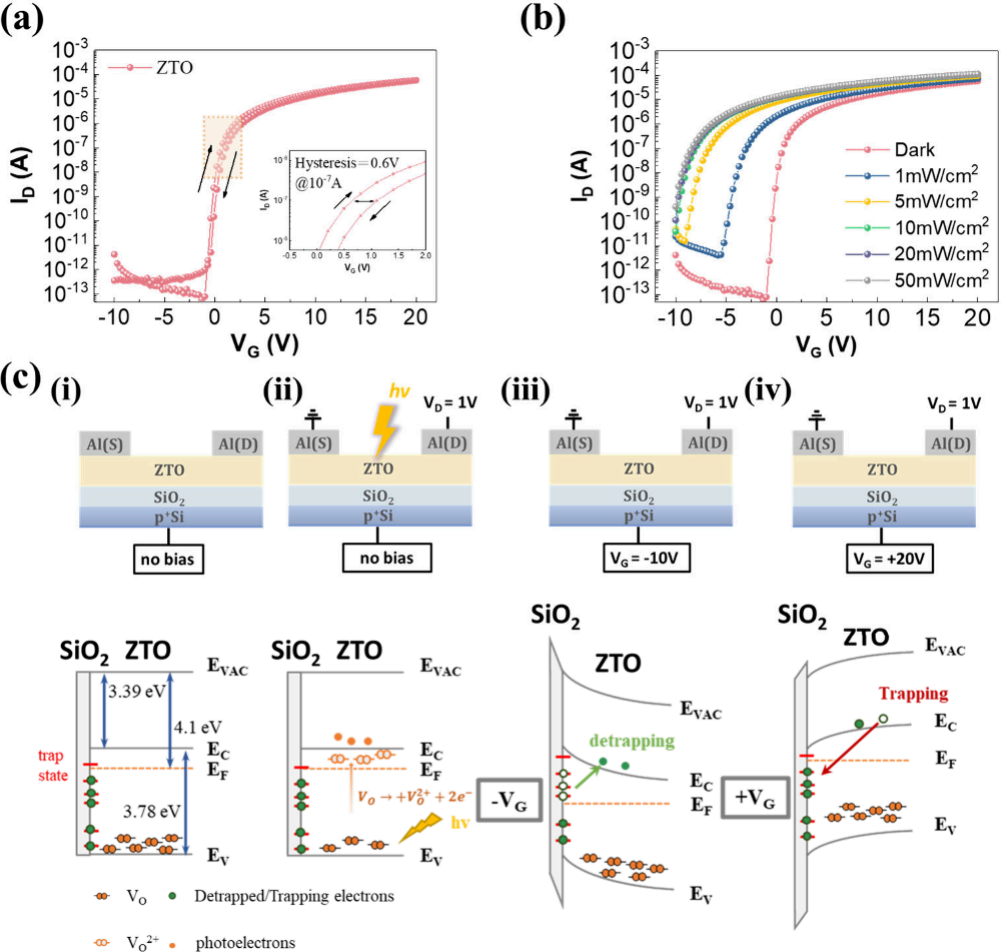
(a) Transfer characteristics of the Al/ZTO/p^+^-Si transistor under dark conditions (*V*_D_ = 1 V). (b) Transfer characteristics of the Al/ZTO/p^+^-Si phototransistor under 405 nm light illumination with various power densities from 1 to 50 mW/cm^2^ at *V*_D_ = 1 V. (c) The schematic illustrates the operational mechanism of the ZTO/SiO_2_ interface, elucidating the trap and detrap of oxygen vacancies during various programming processes. (i) to (iv) Energy band diagrams at the ZTO/SiO_2_ interface during different programming processes, including (i) the initial state (without light stimulation or *V*_G_ bias), during the programming process the state (ii) under light stimulation and (iii) with negative gate bias (*V*_G_ = −10 V) and (iv) with positive gate bias (*V*_G_ = +20 V).

The transfer (*I*_D_–*V*_G_) characteristics of single ZTO phototransistors measured under dark conditions are presented in Figure 2a, respectively. The device swept the gate voltage in the range of −10 to +20 V under a constant 1 V drain-source voltage (*V*_DS_). The *I*_D_–*V*_G_ curve exhibited clockwise hysteresis during the voltage sweep applied to the gate, with a hysteresis window of nearly 0.6 V at *I*_D_ = 10^–7^ A. The presence of defects between the semiconductor and dielectric layers leads to the existence of interface trap sites, which influence the electrical behavior. Therefore, electrons became trapped at the ZTO/SiO_2_ interface, resulting in observed clockwise hysteresis.

To examine the device-to-device variation of the phototransistor, the *I*_D_–*V*_G_ curves and the gate current (*I*_G_) were measured in the dark as shown in Figure S1. The results indicated that the values of *I*_G_ for the ZTO phototransistor were significantly smaller than the drain current (*I*_D_), revealing that the leakage current of the device could be considered negligible. The statistical analyses of the extracted turn-on voltage, hysteresis window, subthreshold swing, and threshold voltage are shown in Table S1, respectively. These statistical results demonstrated that the different ZTO phototransistors were consistent and repeatable.

To evaluate the photoresponsivity performance of the ZTO phototransistor, Figure 2b shows the transfer characteristic curves of the single ZTO phototransistor at *V*_DS_ = 1 V under 405 nm illumination with different power densities (ρ) ranging from 1 to 50 mW/cm^2^. Under 405 nm laser illumination, the turn-on voltage of the ZTO phototransistor shifted toward a more negative direction, and the channel conductance increased as the power densities increased, revealing an increase in carrier density under light illumination.

The operation mechanisms of a single ZTO phototransistor are presented via schematic band diagrams, as shown in Figure 2c. UPS analysis determined the work function (Φ_ZTO_) of ZTO to be 4.1 eV as shown in Figure S3. The difference between *E*_F_ and *E*_V_ in ZTO was determined to be 3.07 eV. The bandgap of ZTO was found to be 3.78 eV, as estimated in Figure 1e. Then, using the formula Φ_ZTO_ – [*E*_g,ZTO_ – (*E*_F,ZTO_ – *E*_V,ZTO_)], the difference between the conduction band and the vacuum band was calculated to be 3.39 eV. Based on these results, the equilibrium band structure can be plotted, as shown in Figure 2c(i).

Figure 2c(ii) illustrates the mechanism of photoelectron generation in the ZTO channel under 405 nm illumination. Neutral oxygen vacancies (V_o_) in the valence band ionized to form positively charged oxygen vacancies (V_o_^2+^), accompanied by the generation of photoelectrons.^25^Figure 2c(iii) showed the ZTO phototransistor under negative gate bias (−*V*_G_). Electrons are trapped at the ZTO/SiO_2_ interface due to trap states. When a negative gate bias (−*V*_G_) was applied, trapped electrons were released, increasing the carrier concentration in the channel. On the other hand, when a positive gate bias (+*V*_G_) was applied, as shown in Figure 2c(iv), electrons were influenced by +*V*_G_ to migrate toward the SiO_2_ interface, where they became trapped at the interface between ZTO and SiO_2_, resulting in a decrease in the drain current.

**Figure 3:**
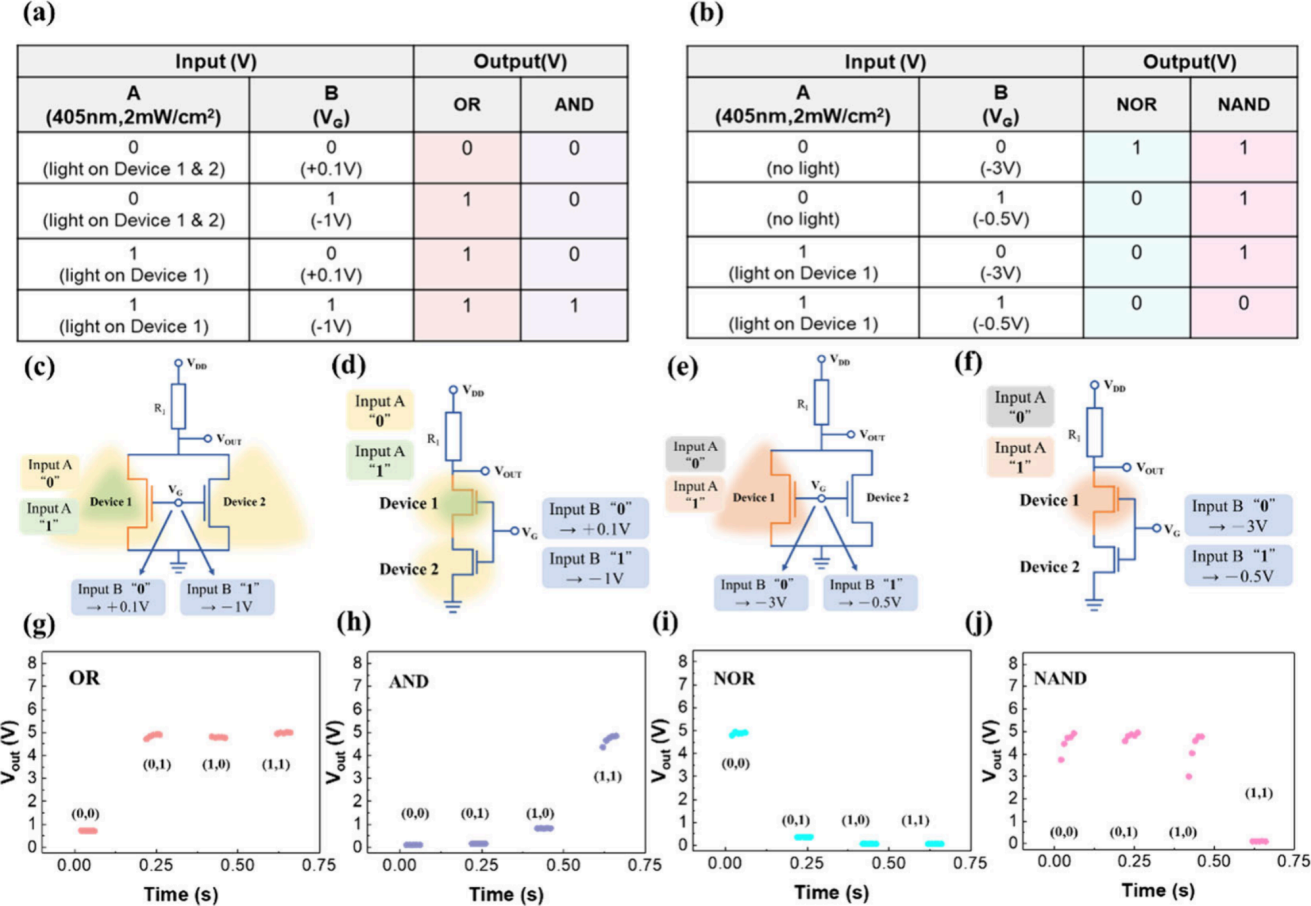
(a) and (b) illustrate the truth tables. (c–f) depict the conditions for the logic gate circuits. A schematic illustration of reconfigurable logic functions is provided. Time-dependent output voltages are shown for the (g) OR gate, (h) AND gate, (i) NOR gate, and (j) NAND gate.

Photonic and electronic logic circuits play a crucial role in advancing integrated circuits, particularly in the development of optoelectronic logic gates.^26,27^ We connected two ZTO phototransistor devices in parallel and series configurations to demonstrate various Boolean logics (OR, AND, NOR, and NAND) by adjusting the input signals. Figure 3a–f illustrated the truth tables and circuit configurations for these logic gates. The schematic diagrams of ZTO TFT in parallel (OR/NOR) and series (AND/NAND) configurations are shown in Figure S4(a),(b). Figure 3a–d illustrated the truth tables and circuit configurations for these logic gates. Additionally, in the circuit diagrams, R_1_ represents a resistor with a resistance of 10 MΩ.

We constructed OR, AND, NOR, and NAND logic operations at a *V*_DD_ of 5 V, as shown in Figure 3g–i. Two different inputs (Input A and Input B) were used for the circuit operation. Input A represented optical pulses applied to the devices, while Input B represented electrical pulses applied to the common gate terminal. The configurations for the OR and AND logic gates are depicted in Figure 3b,c, connected in parallel and series, respectively. In the operation of these two logic gates, for Input A, a logic “0” indicated the application of light pulses (light intensity: 2 mW/cm^2^) to both Device 1 and Device 2, whereas a logic “1” indicated the exclusive application of light pulses to Device 1. For Input B, a logic “0” corresponded to a gate bias of +0.1 V, while a logic “1” corresponded to a gate bias of −1 V.

Figure 3 panels g and h present the *V*_out_ values for the OR and AND gates according to the operation of input signals A and B in the logic gates. As depicted in the OR operation, the input signal (0, 0) for A and B resulted in a V_out_ of “0”, while the input signals (0, 1), (1, 0), and (1, 1) resulted in a V_out_ of “1”. For the AND logic gates, the input signals (0, 0), (0, 1), and (1, 0) for A and B resulted in a V_out_ of “0”, while the input signal (1, 1) resulted in a V_out_ of “1”.

Next, the NOR and NAND logic operations are depicted in Figure 3e,f, respectively. In this operation, for Input A, a logic “0” signified no light pulses to either device, while a logic “1” represented light pulses (light intensity: 2 mW/cm^2^) directed specifically to Device 1. Conversely, for Input B, a logic “0” corresponded to a gate bias of −3 V, while a logic “1” was associated with a gate bias of −0.5 V. Figure 3 panels i and j show the *V*_out_ values for NOR and NAND with different operations of A and B in the logic gates. All of the output states are consistent with the corresponding truth tables for each logic function. In the study, the selection of light illumination and gate voltage (*V*_G_) values was designed to demonstrate the logic operations of OR, AND, NOR, and NAND gates with a hybrid optical-electrical input method. The determination of light intensity (2 mW/cm^2^) and specific gate voltages (+0.1, −1, −3, and −0.5 V) was based on their ability to clearly represent the logic states “0” and “1” within the constructed logic circuits, ensuring that each logic gate operates in accordance with its corresponding truth table. Therefore, these logic gates can serve as fundamental units of integrated circuits, further applicable to programmable logic, thereby enabling integrated circuit computing. To emphasize the uniqueness of our research, we have compiled a comparison table in Table S3 and Table S4. Compared to previous studies, our device utilizes earth-abundant elements (Zn, Sn) and CMOS compatible processes. Our device also employs dual-mode modulation of electricity and light, offering more versatile optoelectronic sensing capabilities. This approach expands its potential in multimodal applications. Additionally, the ZTO device achieves four fundamental Boolean logic gates (AND, OR, NAND, and NOR), utilizing only a single gate terminal. This method significantly simplifies the circuit design, reducing its complexity compared to established methodologies reported in the literature.^28−38^

**Figure 4:**
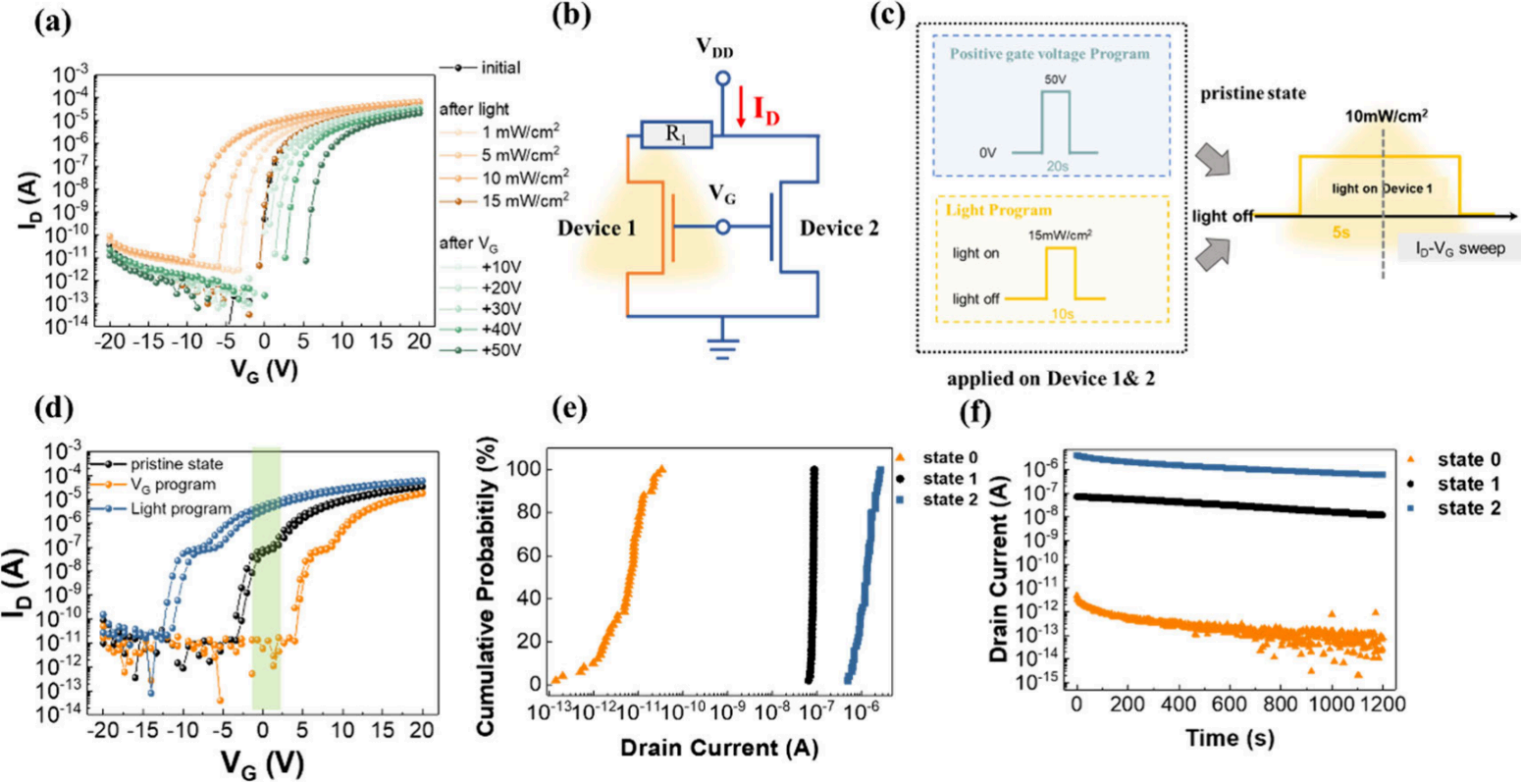
(a) Transfer characteristics of device after light/electric program operations. (b) Schematic diagram of the circuit. (c) Schematic depiction of the pristine state waveform (light intensity: 10 mW/cm^2^) and the operation sequence of optical (light intensity: 15 mW/cm^2^, 10 s) and electrical (*V*_G_ = +50 V, 20 s) programming. (d) Transfer characteristics of programmed phototransistor exhibited three distinct channel conductance states, measured with a fixed drain voltage of 1 V during the gate voltage sweeps. (e) Cumulative probability distribution of drain current levels in each state measurement of 50 cycles. (f) Retention characteristics of each programmed state measured for a duration of 1200 s.

To verify the turn-on voltage shift through long pulse modulation, we examined the transfer characteristics after programming. Figure 4a presents the results of programming the ZTO phototransistor by using both light and electrical methods. During the light programming process, the ZTO phototransistor was exposed to increasing light intensities of 1, 5, 10, and 15 mW/cm^2^, each for a duration of 10 s. For electrical programming, a range of voltages from 10 to 50 V was applied, each for a duration of 20 s. After the device was programmed, *I*_D_–*V*_G_ measurements were conducted on the device. The transfer curves were obtained under both forward and reverse sweeps, with the gate voltage swept from −20 to +20 V at a constant drain voltage of 1 V. Initially, the turn-on voltage was 0 V. After light programming, the turn-on voltage of the ZTO phototransistor shifted to −10 V. This shift is attributed to the increased generation of photoexcited carriers in the ZTO channel with higher light intensity, resulting in an increment in drain current. Conversely, after electrical programming, the turn-on shifted to 5.3 V. The application of a positive bias causes electron accumulation in the ZTO channel, which enhances the trapping of electrons at the ZTO/SiO_2_ interface defects, thereby decreasing the current.

Based on the turn-on voltage adjustable properties of the ZTO phototransistor, we demonstrate the operation of a logic gate, which can present three distinct drain current states in the transfer characteristic curves. Device 1 is connected to a resistor (*R*_1_ = 10 MΩ), which effectively suppresses the current. Initially, a 405 nm light with an intensity of 10 mW/cm^2^ is applied to Device 1 for 5 s, and then *I*_D_–*V*_G_ sweeping is performed. This process is termed the pristine state, as shown in Figure 4c, and the drain current is measured. As illustrated in Figure 4d, the device in the pristine state demonstrates three distinct drain current states, corresponding to the off, intermediate, and on states. Notably, there is an intermediate current state at 10^–8^ A within the *V*_G_ range of −2 to +2 V. Furthermore, we analyzed the current contributions by examining the transport behaviors of the independent sections depicted in Figure 4b. The resulting transfer curves are presented in Figure S5. It can be observed that the first voltage threshold of the parallel circuit closely corresponds with the region of Device 1 in series with R_1_, while the subsequent voltage threshold closely aligns with that of Device 2. Moreover, to clearly illustrate how the devices are connected in Figure 4b, Figure S6 showed the schematic diagram corresponding to the circuit and the actual connection between the ZTO TFT and resistor in Figure 4b.

Before operating in the pristine state, both Device 1 and Device 2 in the circuit diagram can be programmed with light or +*V*_G_, resulting in shifted transfer curves via pulse modulation. These curves exhibit three distinct *I*_D_–*V*_G_ characteristics, represented by blue, black, and orange curves, corresponding to threshold voltages of −12 to +4 and 3 V, respectively. In the pristine state, within the *V*_G_ range of −2 V to +2 V, an intermediate current state around 10^–8^ A is clearly observed, demonstrating a dual-threshold transition. This intermediate state range is advantageous, as it minimizes the repetitive programming results process, facilitating the achievement of target weight values in subsequent measurements. Figure 4e illustrates the cumulative distribution, showing the channel conductance measured after 50 cycles of programming operations. The programming operations result in three distinct current states: state “0”, state “1”, and state “2”, corresponding to “off”, “intermediate”, and “on” states with current values of 10^–12^, 10^–8^, and 10^–6^ A, respectively. In Figure 4f, to understand the retention properties of these three states, the channel conductance was measured and maintained for 1200 s under a drain bias of 1 V, and with *V*_G_ fixed at 0 V. In addition to the on and off states, the intermediate state exhibited a stable current region. This stable intermediate current state enables the representation of more data states compared to binary systems, thereby significantly data storage efficiency. During the measurement process, this state maintains a stable current level, with retention data confirming that these levels remain constant over time without degradation.

**Figure 5:**
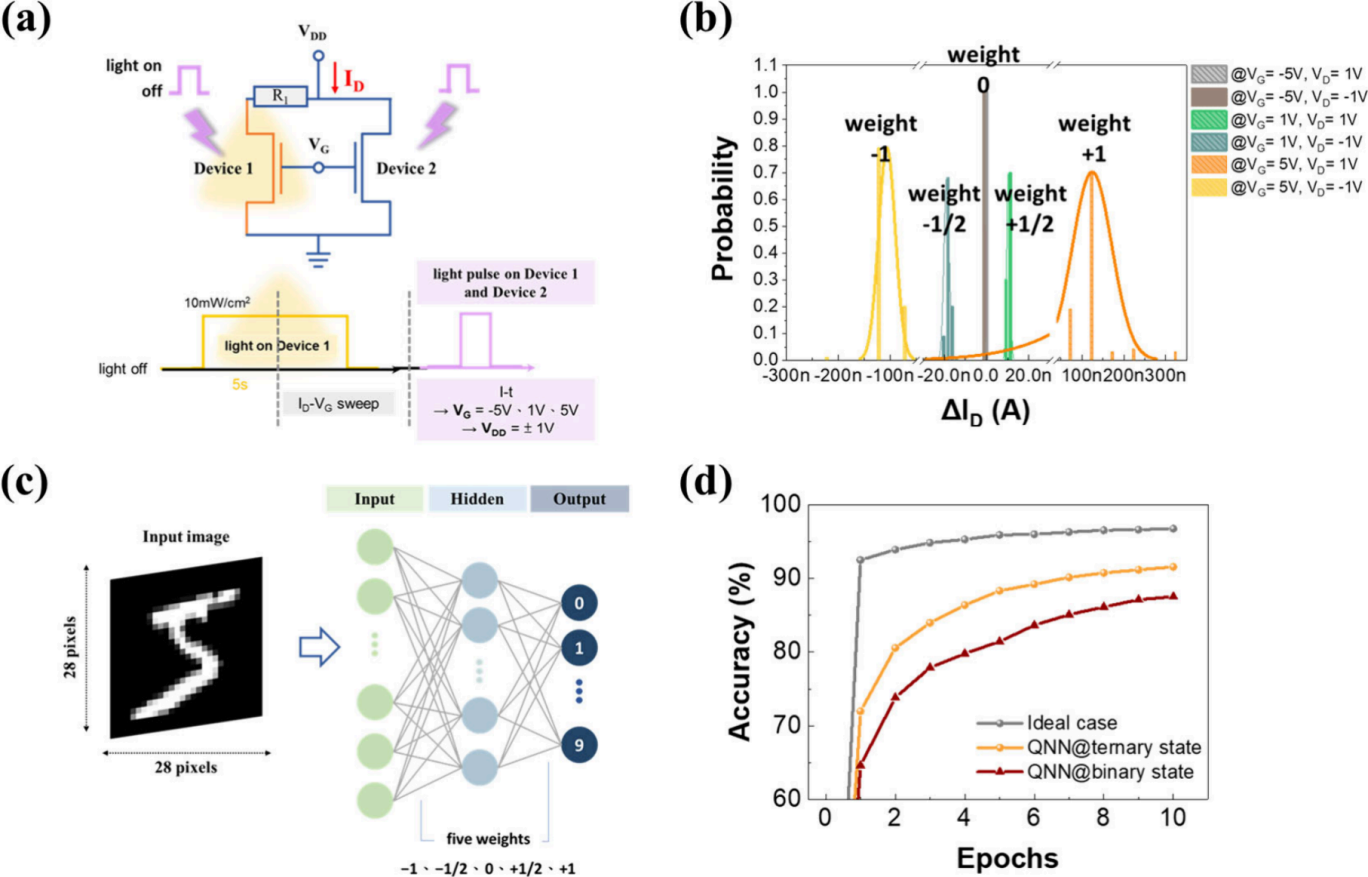
(a) Schematic diagrams of the circuit structure and waveforms. After the pristine state, the depiction under *V*_G_ = −5, +1, +5 V, and *V*_D_ = ±1 V conditions. (b) Probability distribution of the five balanced outputs, derived from the geometric means and standard deviations. (c) Composition of Quantized Neural Network (QNN) layers for image classification. (d) Validation accuracy of Quantized Neural Networks (QNNs) and full-precision Neural Networks models trained on the MNIST data set after 10 training epochs.

As artificial neural network (ANN) computations become increasingly complex, the requirements for storage, computation, and rapid data transfer have significantly increased.^39,40^ Therefore, quantum neural networks (QNNs), which use fewer bits to represent data, were introduced to reduce the number of memory accesses and data transfers. We devised a computational approach for QNNs, providing five quantized weight values, as shown in Figure 5a. By applying positive and negative drain voltages of the same amplitude (*V*_DD_ = ±1 V) to two phototransistors and maintaining a fixed gate bias, we obtained distinct current states were obtained. Following the pristine state operation method depicted in Figure 4c, light pulses were simultaneously applied to both devices. The total output current was then measured, and the photocurrent (Δ*I*_D_) was derived from this measurement.

Our measurements demonstrated the positive and negative weight values (−1, – 1/2, 0, 1/2, and 1) in the QNN, as illustrated in Figure 5b. The mean (μ) and geometric standard deviation (σ_g_) for each weight value from −1 to +1 were calculated, as shown in Table S5, with values of (−1.05 × 10^–7^, 1.11704), (−1.89 × 10^–8^, 1.06334), (6.97 × 10^–10^, 1.17942), (1.04 × 10^–8^, 1.07881), and (1.18 × 10^–7^, 1.32198), respectively. These measured data were used to simulate image classification, achieving the five quantized weight values for QNN operations by using a pair of phototransistor devices.

Next, we evaluated the performance through the image classification process using the MNIST data set. Figure 5c schematically illustrates a neural network structure with 784 input neurons, 256 hidden neurons, and 10 output neurons, corresponding to the 10-digit classes from 0 to 9. The training utilized the five measured quantized values. For comparison, we performed the same image classification task using a conventional, full-precision neural network.

Figure 5d presents the validation accuracy of the full-precision neural network and the QNN model in the image classification tasks with the validation accuracy evaluated at each training epoch. After training for 10 epochs, the accuracy achieved by the full-precision neural network and the QNN model was 96.7% and 91.6%, respectively. Note that to incorporate the device characteristics into this model, we introduced the concept of Gaussian probability distribution and set the measured current mean values with the corresponding standard deviation to the five quantize weights, as the basis for QNN weight quantization. If the neural network merely employs five discrete weights by dividing the range [−1, 1] into 5 intervals, without incorporating any probability distribution, the resulting accuracy is lower than 60%. Although the accuracy of our QNN model is not as high as that of full-precision ANN models, our approach ultimately aims to reduce computational complexity while maintaining reasonable accuracy. Besides, our QNN model indicates that even with fewer weights, our accuracy is comparable to established results in QNN research.^22^ Additionally, conducting the same operations under binary conditions produced three weight values. The network was trained using an identical architecture for 10 epochs, resulting in an accuracy of 87.5%. Implementing ANN algorithms requires substantial storage, powerful computing units, and sufficient energy to support significant data transfers between memory and computing units. Therefore, QNN algorithms, which use fewer bits to represent data, have been introduced to reduce the level of memory access and data transfer, further minimizing energy consumption.

## 3 Conclusion

In summary, we successfully utilized a configuration of two zinc–tin oxide (ZTO) phototransistors to demonstrate fundamental Boolean logic gates, including AND, OR, NAND, and NOR, by applying gate bias and optical pulse input signals. Additionally, by modulating the turn-on voltage of a single device via light illumination, we achieved binary and ternary logic states. Furthermore, by utilizing these ternary logic states, we achieved discrete and balanced quinary current levels. The implemented quantized quinary weights were utilized to execute the QNN algorithm. Software demonstrations revealed that the QNN algorithm based on the MNIST data set achieved a classification accuracy of 91.6%, while the main scope of this work was to demonstrate that our ZTO transistor circuit could achieve three current states and be further applied to QNN. The efficiency of the QNN was certainly not perfect, and it should be optimized in our future study. Above all, these results indicated the potential for significant advancements in the Boolean logic gate and energy-efficient artificial intelligence and in-memory computing applications.

## 4 Experimental Section

### Fabrication of ZTO Phototransistor

The semiconductor film was fabricated by using a precursor solution of ZTO. Zinc acetate dihydrate (Zn (CH_3_COO)_2_·H_2_O) and tin chloride dihydrate (SnCl_2_·2H_2_O) were used as precursors (molar ratio of Zn:Sn = 1:1). The ZTO solution was then mixed with 2-methoxyethanol solvent and stirred using a magnetic stirrer for 20 h to ensure chemical homogeneity. Subsequently, the precursor solutions were spin-coated onto a p^+^-type Si wafer with a SiO_2_ layer (100 nm thickness) at 4000 rpm for 60 s, followed by annealing at 500 °C for 1 h in air. The patterning process for the solution-processed ZTO is as follows: First, coat the sample with HMDS, then apply the S1813 photoresist. After baking at 115 °C, it was exposed under a photomask in UV light, followed by development in TMAH solution. Once the sample was rinsed with DI water, it was then etched using a 1:2 nitric acid and water solution. In the end, the photoresist was removed with acetone, and the sample was rinsed with DI water and dried with a nitrogen gun, resulting in the patterned ZTO. Finally, the Al source and drain electrodes (150 nm thick) were evaporated via E-beam evaporation through a shadow mask to define the phototransistor channel width (2000 μm) and length (80 μm).

### Material Characterization

The film thickness and EDS elemental mapping analysis were obtained by using a field emission transmission electron microscope (JEM-2100F, JEOL) with an acceleration voltage of 200 keV. The absorption spectrum of the ZTO was collected by using a UV–VIS/NIR spectrophotometer (UH5700, Hitachi). The chemical bonding states of the oxygen atoms in the ZTO films were characterized by X-ray photoelectron spectroscopy (XPS, JEOL JAMP-9500F).

### Electrical and Optoelectronic Characterization

The electrical and optoelectronic characteristics of the ZTO phototransistor were characterized using a Keysight 81150A and an Agilent 4156C semiconductor parameter analyzer in an ambient atmosphere in a dark box. For light illumination, two 405 nm lasers (SDL-405-LM-010T and SONY SLD3232VF) were employed as light sources during the optoelectronic measurements. We used a pulse function arbitrary noise generator (Keysight 81150A) to accurately control the on–off switch of the lasers during the measurement. The intensity of the laser source was measured using a laser power meter (Model 843-R, Newport).

### Neural Network Simulation

To validate the efficacy of QNN, software simulations were performed within the Python programming environment. The MNIST data set composed of 28 × 28 pixel images was used with a training set of 60,000 images followed by a test set of 10,000 images for evaluation and recognition. The input layer of the network consisted of 784 neurons, each corresponding to a pixel in the flattened image data. The hidden layer comprised 256 neurons, facilitating stable and efficient computation throughout the training process. Finally, the output layer consisted of 10 neurons, each corresponding to one of the ten classes in the data set.

## Supporting Information Available

Device-to-device and cycle-to-cycle variations in *I*_D_–*V*_G_ characteristics under dark conditions (*V*_D_ = 1 V); turn-on voltage, hysteresis window, S.S. and threshold voltage values for device-to device and batch-to-batch variations; XPS spectra; integrated area of the XPS spectrum; UPS spectrum of ZTO; circuit diagrams; photo of the ZTO TFT devices; comparative analysis of Boolean logic operations in recently reported work; table of geometric mean and geometric standard deviation of five weight (PDF)
