# Supplementary material for: Interfacial Electronic Charge Trapping and Photonic Carrier Excitation Coupling in Solution-Processed Zinc–Tin Oxide Thin-Film Transistors Applied for Logic Gate Design and Quantized Neural Network
Source: ACS Appl Mater Interfaces. 2024 Dec 18;17(1):1477–84. doi: 10.1021/acsami.4c15102 (PMC11783503; doi:10.1021/acsami.4c15102)
Supplement: Supplementary file 1 — am4c15102_si_001.pdf [file am4c15102_si_001.pdf]

Supporting Information

Interfacial Electronic Charge Trapping and Photonic Carrier Excitation Coupling in Solution-Processed Zinc-Tin Oxide Thin-Film Transistors Applied for Logic Gate Design and Quantized Neural Network

Pei-Hsuan Chang[1], Wun-Yun Lin[1], Ya-Chi Huang[1], Yu-Chieh Chen[1], Li-Chung Shih[1], Jen-Sue Chen[1,2]*

1 Department of Materials Science and Engineering, National Cheng Kung University, Tainan 70101, Taiwan

2 Academy of Innovative Semiconductor and Sustainable Manufacturing, National Cheng Kung University, Tainan 70101, Taiwan

*Corresponding author. E-mail: jenschen@mail.ncku.edu.tw

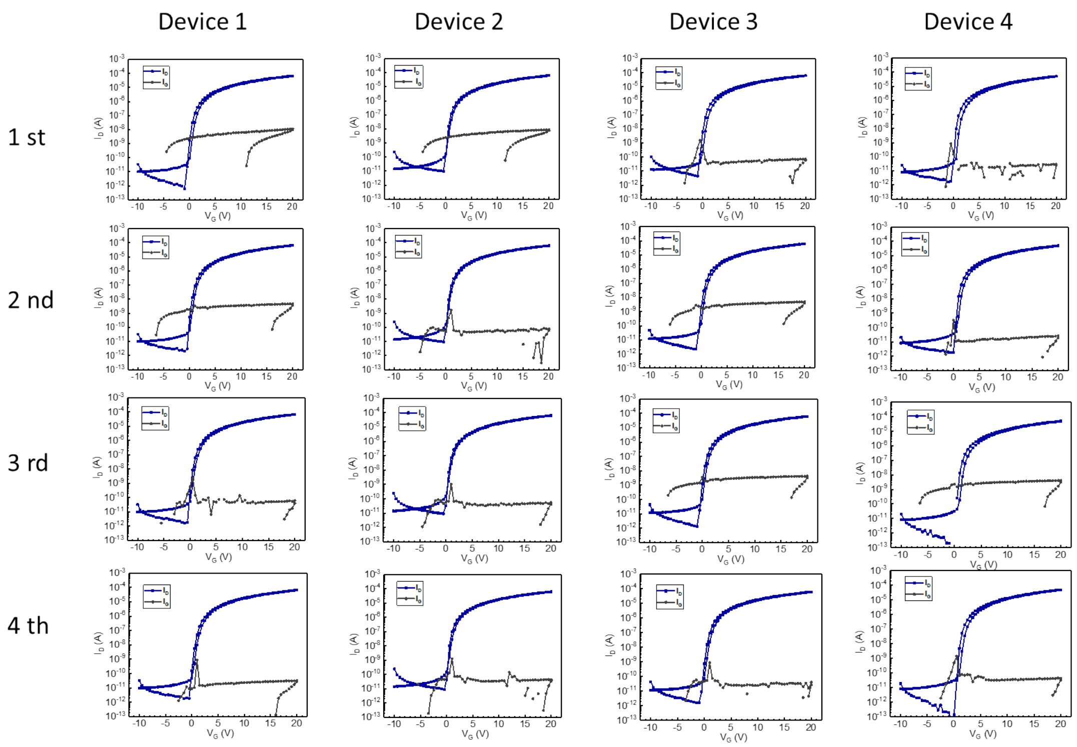

Figure S1. Device-to-device and cycle-to-cycle variations in I_D-V_G and I_G-V_G characteristics under dark conditions (V_D=1 V).

Table S1. Turn-on voltage, Hysteresis window, S.S. and Threshold voltage values for device-to device and batch-to-batch variations

Batch A	Device 1	Device 2	Device 3	Device 4	Average	STD.
V_{on} (V)	-1	-0.5	-1	-0.5	-0.75	0.288675
Hysteresis window (V)	0.5	0.5	0.5	0.5	0.5	0
S.S. (V/dec)	0.29	0.3	0.32	0.27	0.295	0.020817
Threshold voltage (V)	5.7	5.71	5.45	5.84	5.675	0.162993
**Batch B**	**Device 1**	**Device 2**	**Device 3**	**Device 4**	**Average**	**STD.**
V_{on} (V)	-0.5	-0.5	-1	0	0.5	0.408248
Hysteresis window (V)	0.5	0.5	0.5	0.5	0.5	0
S.S. (V/dec)	0.28	0.32	0.34	0.25	0.297	0.040311
Threshold voltage (V)	5.78	5.79	5.66	6.19	5.855	0.231012
**Batch C**	**Device 1**	**Device 2**	**Device 3**	**Device 4**	**Average**	**STD.**
Von (V)	-0.5	-0.5	-1	-0.5	0.625	0.25
Hysteresis window (V)	0.5	0.5	0.5	0.5	0.5	0
S.S. (V/dec)	0.27	0.29	0.33	0.36	0.312	0.040311
Threshold voltage (V)	5.85	5.92	5.71	6.12	5.9	0.170685
**Batch D**	**Device 1**	**Device 2**	**Device 3**	**Device 4**	**Average**	**STD.**
Von (V)	-0.5	-0.5	-1	-0.5	0.625	0.25
Hysteresis window (V)	0.5	0.5	0.5	0.5	0.5	0
S.S. (V/dec)	0.29	0.3	0.32	0.27	0.295	0.020817
Threshold voltage (V)	5.92	5.59	5.78	6.29	5.9	0.296029

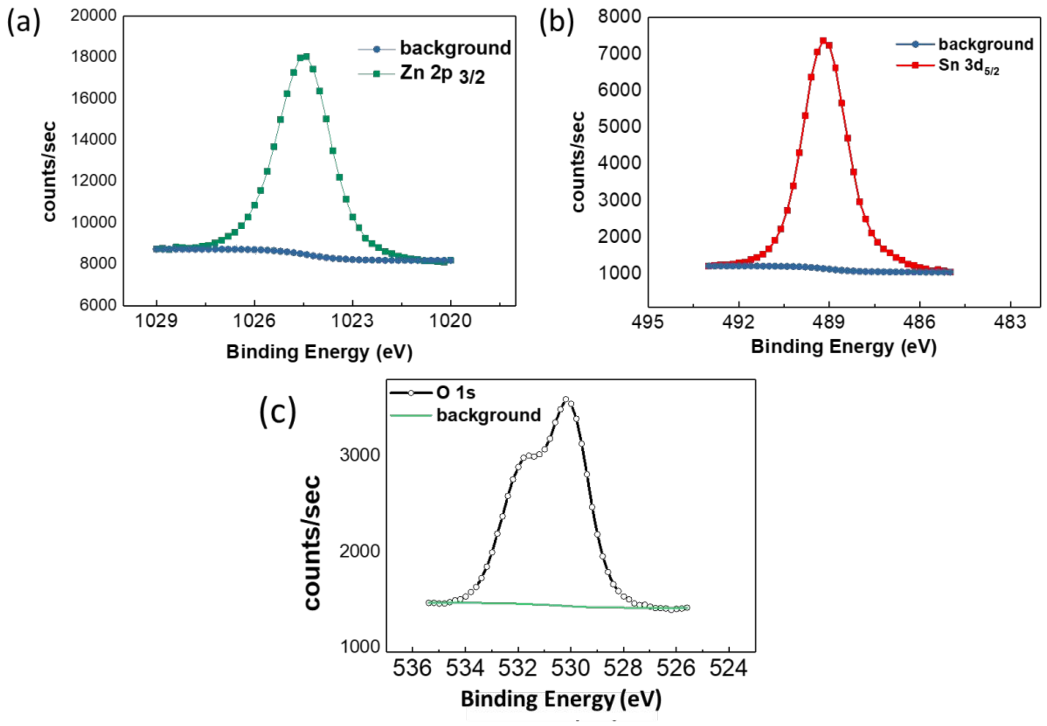

Figure S2. The XPS spectra of (a)Zn $2p_{3/2}$, (b)Sn $3d_{5/2}$, and (c)O 1s core levels.

Table S2. The integrated area of the XPS spectrum and sensitivity factor for each element.

**Elements /electron orbit**	**Integrated Area**	**Sensitivity factor**
**Zn** $2p_{3/2}$	**20796.9**	**18.92**
**Sn** $3d_{5/2}$	**12369**	**14.8**
**O** 1s	**6523.0**	**2.93**

Atomic ratio = $(A_{area,A}/F_A)$: $(A_{area,B}/F_B)$: $(A_{area,C}/F_C)$ (equation S1)

$$\therefore \ Zn : Sn : O = \frac{A_{Zn\ 2p3/2}}{F_{Zn\ 2p3/2}} : \frac{A_{Sn\ 3d5/2}}{F_{Sn\ 3d5/2}} : \frac{A_{O\ 1s}}{F_{O\ 1s}} = \frac{20796.9}{18.92} : \frac{12369.0}{14.8} : \frac{6523}{2.93}$$

= **1.3 : 1 : 2.7**

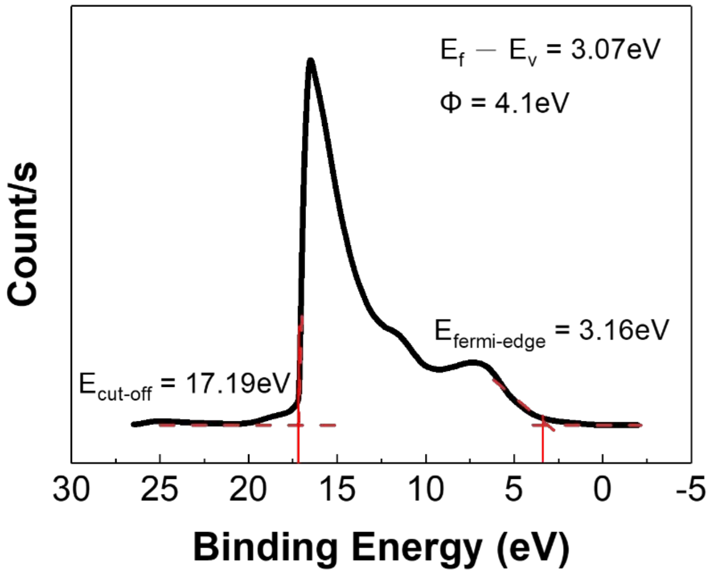

Figure S3. UPS spectrum of the ZTO thin film.

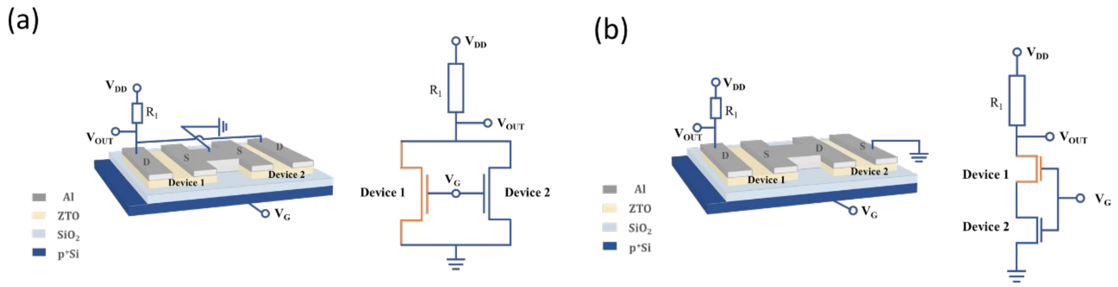

Figure S4. (a) Schematic diagram of the ZTO TFT in parallel logic circuit (OR/NOR) (b) Schematic diagram of the ZTO TFT in series logic circuit (AND/NAND).

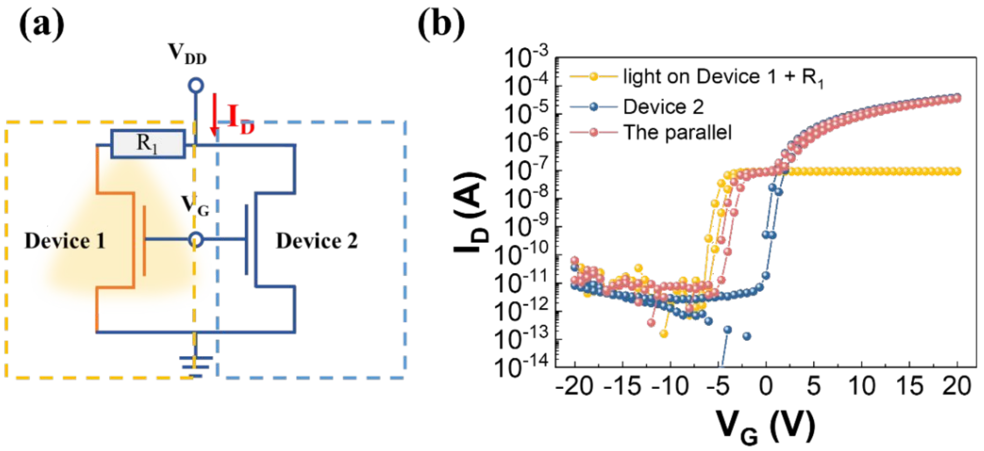

Figure S5. (a)The circuit diagram is schematically illustrated, where a 10 MΩ resistor (b) Current transfer curve.

(a)

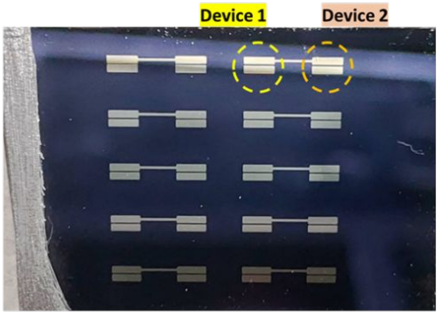

(b)

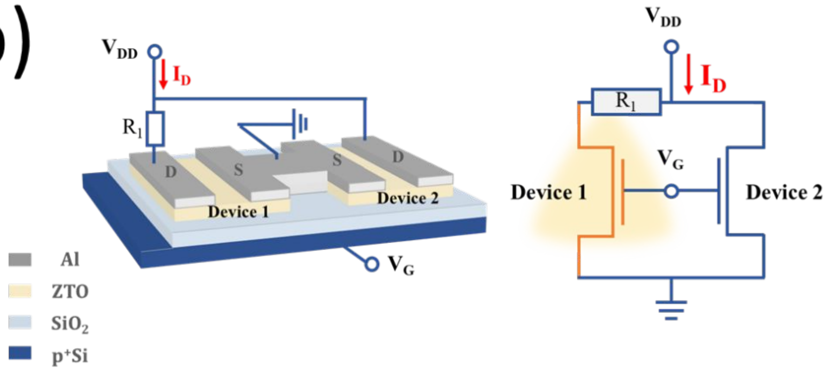

(c)

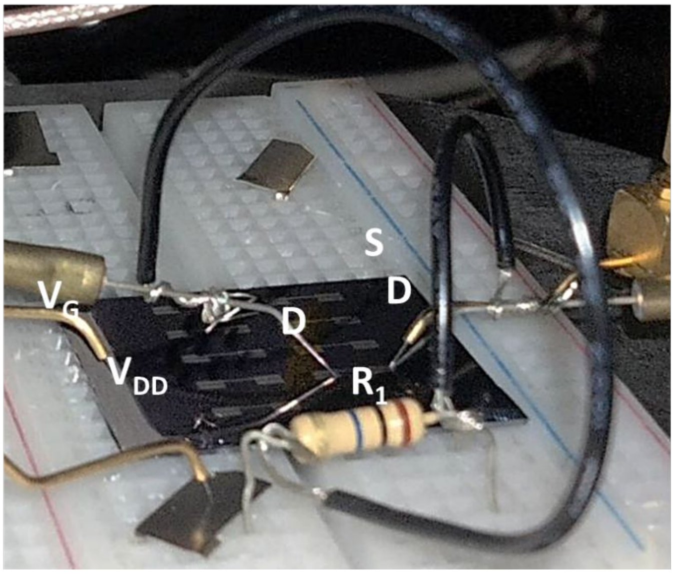

Figure S6. (a) The photo of the ZTO TFT devices and (b) the schematic diagram corresponding to the circuit shown in Figure S5. (c) The photograph of the actual connection setup.

Table S3. A comparative analysis of Boolean logic operations in recently reported works.

Device structure	Modulation strategy	Components	Gate Counts	Supplied voltage (V_{DD})	Pulse width	Logic Type	Ref.
**Al/ZTO/SiO_2/Si**	**Electricity + light (405nm)**	**2 transistors, 1 resistor**	**1**	**5V**	**100ms**	**AND,OR, NAND, NOR**	**This work**
**Au/29DPP-TT/Al_2O_3/Si**	Electricity	3 transistors	2	20V	20s	NAND, NOR	[1]
**Al/Al_2O_3/pEGDMA/SiO_2/Si**	Electricity	2 transistors, 1 resistor	2	1V	2.5s	NAND, NOR	[2]
**Au/hBN/BP/MoS_2/Si_3N_4/Si**	Electricity	1 transistor	2	NA	2s	AND, OR, NAND, NOR, XOR, and XNOR	[3]
**Au/MoS_2/h-BN/graphene/SiO_2/Si**	Electricity + light(532nm)	1 transistor	1	NA	500ms	AND, OR, NAND, NOR	[4]
**Au/WSe_2/$LiNbO_3$/SiO_2/Si**	Electricity	2 transistors, 2 resistors	2	5V	500ms	AND, OR	[5]
**ITO/IGZO/Pectin/ITO/glass**	Electricity	1 transistor, 1 resistor	4	1V	--	AND, OR	[6]
**Ni/ITO/HfLaO/W/SiO_2/Si**	Electricity	1 transistor	1	NA	--	AND, OR	[7]

Table S4. The device performance comparison of oxide-based TFTs applied in Boolean logic gates.

Device structure	Modulation strategy	Components	Supplied voltage (V_{DD})	I_{OFF} (A)	On/off ratio	Logic gate circuits	Ref.
**Al/ZTO/SiO_2/Si**	**Electricity + light (405nm)**	**2T1R**	**1V**	$\mathbf{10^{-12}}$	$\mathbf{10^{7}}$	**AND,OR, NAND, NOR**	**This work**
**ITO/IGZO/Pectin/ITO /glass**	Electricity	1T1R	1V	10^{-10}	10^{6}	AND,OR	[6]
**Ni/ITO/HfLaO/W/SiO_2/Si**	Electricity	1T	1V	10^{-11}	10^{6}	AND,OR	[7]
**PEDOT:PSS/CSPE/In_2O_3 /ITO/glass**	Electricity	3T	1V	10^{-9}	10^{6}	NAND,NOR XOR	[8]
**P3-SWNT/PDMS/$BaTiO_3$ /sSWCNT/PDMS**	Electricity	2T	10V	10^{-10}	10^{4}	NAND, NOR	[9]
**Polymer resist/Cr/Au/IGZO/CNTs /Al_2O_3/SiOx/Al**	Electricity	4T	5V	10^{-10}	10^{5}	NAND,NOR	[10]

Table S5. Geometric mean and Geometric standard deviation of five weight.

Outputs (weights)	Geometric mean	Geometric standard deviation
-1	-1.05×10^{-7} A	1.11704
-1/2	-1.89×10^{-8} A	1.06334
0	6.97×10^{-10} A	1.17942
+1/2	1.04×10^{-8} A	1.07881
+1	1.18×10^{-7} A	1.32198

References

(1) Hong, J.; Kim, J.; Li, Z.; Cong, C.; Rand, B. P.; Nam, S. Y.; Kim, S. H.; Kim, Y.-H. Facile Direct Printing of DPP-Based Polymers for Organic Field-Effect Transistors and Logic Gates. *ACS Applied Electronic Materials* **2023,** *5* (8), 4114-4124.

(2) Yu, J.-M.; Lee, C.; Han, J.-K.; Han, S.-J.; Lee, G.-B.; Im, S. G.; Choi, Y.-K. Multi-functional logic circuits composed of ultra-thin electrolyte-gated transistors with wafer-scale integration. *Journal of Materials Chemistry C* **2021,** *9* (22), 7222-7227.

(3) Sang, W.; Xiang, D.; Cao, Y.; Tan, F.; Han, Z.; Songlu, W.; Zhou, P.; Liu, T. Highly Reconfigurable Logic-In-Memory Operations in Tunable Gaussian Transistors for Multifunctional Image Processing. *Advanced Functional Materials* **2024,** *34* (4), 2307675.

(4) Liu, X.; Wang, Z.; Huang, H.; Liu, C.; Niu, W.; Xie, Z.; Hao, D.; Fu, H.; Liu, X.; Zou, X. Microneural Network System Based on MoS2/h-BN/Graphene van der Waals Heterojunction Transistor. *ACS Applied Nano Materials* **2023,** *6* (17), 16046-16054.

(5) Tong, L.; Peng, Z.; Lin, R.; Li, Z.; Wang, Y.; Huang, X.; Xue, K.-H.; Xu, H.; Liu, F.; Xia, H. 2D materials–based homogeneous transistor-memory architecture for neuromorphic hardware. *Science* **2021,** *373* (6561), 1353-1358.

(6) Guo, J.; Liu, Y.; Zhou, F.; Li, F.; Li, Y.; Huang, F. Linear Classification Function Emulated by Pectin-Based Polysaccharide-Gated Multiterminal Neuron Transistors. *Advanced Functional Materials* **2021,** *31* (33), 2102015.

(7) Liu, Y.; Wang, T.; Xu, K.; Li, Z.; Yu, J.; Meng, J.; Zhu, H.; Sun, Q.; Zhang, D. W.; Chen, L. Low-power and high-speed HfLaO-based FE-TFTs for artificial synapse and reconfigurable logic applications. *Materials Horizons* **2024,** *11* (2), 490-498.

(8) Marques, G. C.; Birla, A.; Arnal, A.; Dehm, S.; Ramon, E.; Tahoori, M. B.; Aghassi-Hagmann, J. Printed logic gates based on enhancement-and depletion-mode electrolyte-gated transistors. *IEEE Transactions on Electron Devices* **2020,** *67* (8), 3146-3151.

(9) Cai, L.; Zhang, S.; Miao, J.; Yu, Z.; Wang, C. Fully printed stretchable thin-film transistors and integrated logic circuits. *ACS nano* **2016,** *10* (12), 11459-11468.

(10) Honda, W.; Arie, T.; Akita, S.; Takei, K. Mechanically flexible and high-performance CMOS logic circuits. *Scientific reports* **2015,** *5* (1), 15099.
